# Idiopathic Sclerosing Encapsulating Peritonitis: A Rare Cause of Subacute Intestinal Obstruction

**DOI:** 10.1155/2016/8206894

**Published:** 2016-08-25

**Authors:** Mei Chin Lim, Niketa Chandrakant Chotai, Danilo Medina Giron

**Affiliations:** ^1^Department of Diagnostic Radiology, Tan Tock Seng Hospital, 11 Jalan Tan Tock Seng, Singapore 308433; ^2^Department of Pathology, Tan Tock Seng Hospital, 11 Jalan Tan Tock Seng, Singapore 308433

## Abstract

We present a case of a previously healthy 50-year-old gentleman who had recurrent vomiting and abdominal pain of two-month duration. The patient was subsequently diagnosed with abdominal cocoon on computed tomography. Idiopathic sclerosing encapsulating peritonitis, also known as abdominal cocoon, is a rare cause of small bowel obstruction. Visualization of variable encasement of the small bowel loops by a characteristic membranous sac, either preoperatively with cross-sectional imaging or intraoperatively, is the key to diagnosis. This is a highly treatable condition; surgical excision of the sac with adhesiolysis facilitates a full recovery in affected patients.

## 1. Introduction

Sclerosing encapsulating peritonitis (SEP) is an uncommon cause of subacute small bowel obstruction. SEP is classically described as a partial or total encasement of the small intestines by a fibrotic sac-like membrane [1, 2]. There is no known aetiology for this enigmatic condition, and it can be broadly divided into two forms, primary which is idiopathic in nature (also called abdominal cocoon) and secondary which has been associated with various localized and systemic processes [3–6].

We describe a case of idiopathic SEP in an adult gentleman that presented with symptoms of intermittent intestinal obstruction. In this report, we seek to highlight the pertinent radiological, surgical, and histopathological characteristics that facilitate the diagnosis and review the current literature on this rare condition. A potential mimic for this condition, midgut volvulus, will be briefly discussed.

## 2. Case Report 

A 50-year-old Chinese man presented with recurrent episodes of vomiting, diarrhoea, and abdominal distension over two months. He had no significant medical, surgical, or family history of note. The patient was afebrile and haemodynamically stable. Physical examination revealed a vague central abdominal mass that was soft and nontender on palpation. There was no evidence of peritonitis and bowel sounds were normal. Initial laboratory investigations showed mildly elevated white cell count of 12.6 × 10^9^/L (reference range 3.6–9.3 × 10^9^/L) and C-reactive protein level of 18.5 (reference range 0.0–0.5). Tumour markers were not raised.

**Figure 1:**
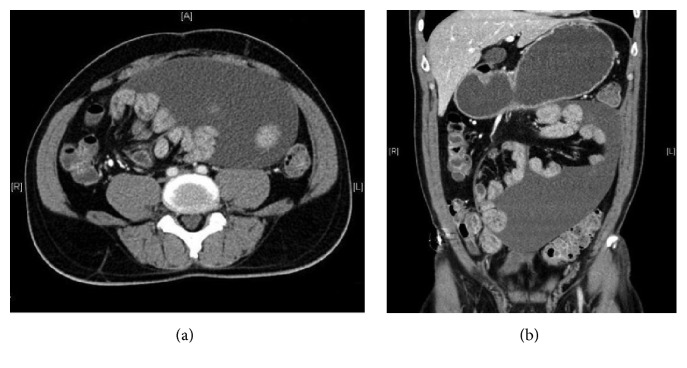
Contrast-enhanced CT in (a) axial and (b) coronal sections showing encasement of the mildly dilated small bowel loops within a membranous sac with thin wall. Moderate amount of free fluid is noted within the sac.

Abdominal radiographs performed showed no dilated bowel loops. A contrast-enhanced computed tomography (CT) scan was subsequently performed, which demonstrated generalized mild dilatation of the small bowel loops. There was total encasement of the small bowel loops by a thin membrane of imperceptible wall within a fluid-filled sac (Figure 1). There was no imaging evidence of small bowel ischemia. No significant inflammatory fat stranding or lymphadenopathy was detected. Diagnosis of an abdominal cocoon was suggested.

The patient underwent explorative laparotomy, which found multiple dilated small bowel loops enclosed within a fluid-filled membranous sac. Interloop adhesions were seen extending from the duodenojejunal junction to the ileocecal junction. Cystostomy of the sac and adhesiolysis were subsequently performed, with decompression of the dilated bowel loops. Straw-colour fluid was noted on cystotomy.

**Figure 2:**
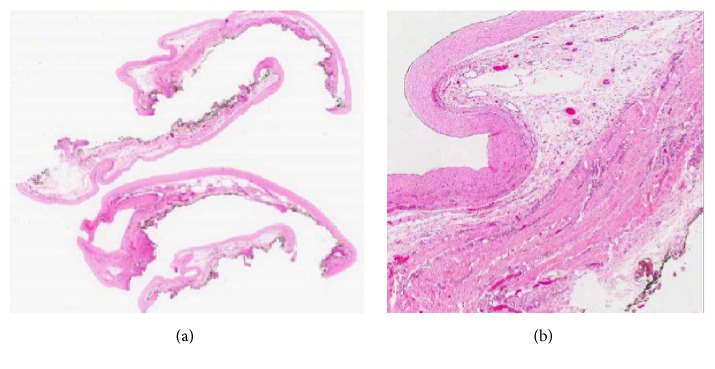
Histology of the cyst wall in (a) 0.3 times magnification and (b) 2 times magnification featuring a fibrocollagenous wall and underlying loose connective tissue.

Histology of the sac showed membranous soft tissue featuring a fibrocollagenous wall with no apparent epithelial or mesothelial lining. Patchy acute and chronic inflammation was noted (Figure 2). There was no evidence of cellular atypia or malignancy. Cytology of the peritoneal fluid revealed no malignant cells.

The patient experienced prolonged postoperative ileus. He was managed conservatively and progressive recovery was observed. The patient had an uneventful discharge on postoperative day 26. One-year follow-up of the patient revealed no evidence of recurrence.

## 3. Discussion

Idiopathic or primary SEP, also known as abdominal cocoon, is first described by Foo et al. in 1978. It was initially reported to typically affect adolescent girls from the tropical or subtropical climate and postulated retrograde menstruation as a cause for the condition [1]. However, subsequent case reports of older male subjects in nontropical countries refute this hypothesis [2].

Secondary SEP is a more common form and is most frequently associated with continuous ambulatory peritoneal dialysis (CAPD) [3]. About 1% of CAPD patients may be inflicted with SEP, with the risk increasing with years of peritoneal dialysis [4]. Failure of ultrafiltration, loss of weight, and symptoms of recurrent intestinal obstruction should prompt the exclusion of SEP [5].

Other less frequent associations include ventriculoperitoneal or peritoneal-venous shunting, prolonged consumption of beta-blocker (particularly practolol), and, rarely, postliver transplantation, tuberculosis, sarcoidosis, gastrointestinal malignancy, ovarian thecoma, endometriosis, presence of fibrogenic foreign body, and postpenetrating abdominal injury [5, 6].

SEP is an unusual cause of subacute small bowel obstruction. Although the pathogenesis is largely obscured, SEP is a benign and highly treatable condition. Diagnosis may be initially suspected in a patient with recurrent episodes of vomiting, diarrhoea, abdominal pain, or distension which may resolve spontaneously. A nontender abdominal mass may be palpated during physical examination.

Abdominal radiographs are generally nonspecific and may demonstrate dilated small bowel loops with air-fluid levels. Normal radiographs may also be encountered, such as in our case.

CT scan, being one of the common requested investigations in patients with suspected intestinal obstruction, will facilitate clinching the diagnosis upon visualization of characteristic encasement of variable length of the small bowel loops in the central abdomen within a membrane-like sac, causing small bowel obstruction [7–10]. Other findings are nonspecific and include peritoneal thickening, peritoneal calcification, fixation of intestinal loops, free fluid, and reactive lymphadenopathy [7]. Other useful information that can be obtained from CT includes the extent of bowel involvement, status of involved bowel loops, and any associated complications.

On barium meal and follow-through, the appearance of a conglomerate of multiple small bowel loops giving rise to a “cauliflower” configuration is described [5]. Transit time of contrast is usually prolonged. In practical scenario, barium studies are generally nonspecific for this condition and are not routinely performed [8].

The definite diagnosis is made during exploratory laparotomy or laparoscopy [11, 12]. A simple surgical release of the entrapped bowels via removal of the fibrotic membrane with adhesiolysis is the suggested treatment for this condition [13]. Bowel resection is usually not required if there are no signs of ischemia in the bowel. The majority of patients with idiopathic SEP have excellent prognosis and generally do well following surgery, with low rates of complication [14]. These rare postoperative complications include adhesions, infection, and formation of enterocutaneous fistula [11, 14]. On the other hand, prognosis for secondary forms of SEP tends to be worse. In particular, patients with peritoneal dialysis-associated SEP have one-year mortality rate of 30% to 57% [15, 16].

Histology of the excised membrane characteristically shows fibrin deposits, thought to be as a result of increase exudation of plasma contents from the small vessels of the peritoneum [17]. Associated inflammatory cell components are often seen. Conservative treatment with drugs such as tamoxifen, corticosteroids, and immunosuppressants has been proposed in asymptomatic patients or in mild nonobstructive cases, although limited literature is available [11, 18].

**Figure 3:**
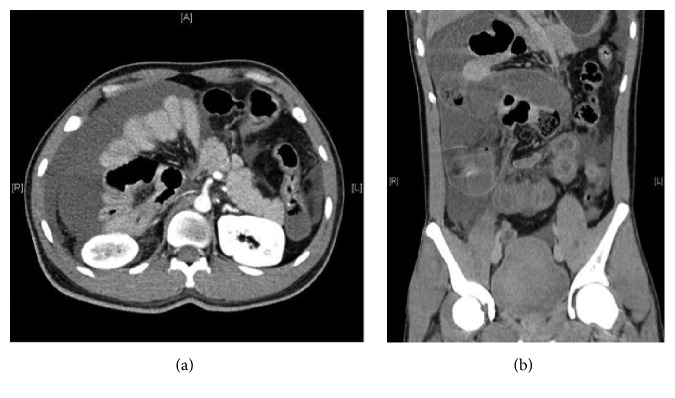
Contrast-enhanced CT in (a) axial and (b) coronal sections showing clustering of dilated small bowel loops in the right side of the abdomen, with no visualization of an encapsulating sac. The superior mesenteric artery is to the right of the superior mesenteric vein (a).

We also present a rare case of midgut volvulus in a 31-year-old man for comparison (Figure 3). Midgut malrotation is a congenital anomaly in rotation of the foetal intestine during embryological development. The vast majority of patients present in the first month of life, with 90% presenting by 1 year of age. Midgut volvulus, which is a serious complication of malrotation, is seldom encountered in adulthood [19]. In this case, there is clustering of mildly dilated small bowel loops on the right side of the abdomen, with twisting of the mesentery around the axis of the mesenteric vessels. There is reversal of the normal relation of the superior mesenteric vessels, that is, the superior mesenteric artery (SMA) to the right of the superior mesenteric vein (SMV), resulting in a characteristic “whirlpool appearance” [20]. No membranous sac is detected. The major distinguishing factors in SEP are the visualization of the characteristic membranous sac and the preserved relationship of SMA and SMV.

## 4. Conclusion

Abdominal cocoon, though rare, is a benign and highly treatable condition. It should be considered in the differential diagnoses in patients presenting with symptoms of intermittent bowel obstruction. In the past, diagnosis was generally made intraoperatively upon visualization of an encapsulating membranous sac which is adherent to underlying bowel loops. With improving resolution of CT imaging, preoperative imaging can now help clinch the diagnosis. A high index of suspicion is critical to make the correct diagnosis.
